# Exosomes as a Cell-free Therapy for Myocardial Injury Following Acute Myocardial Infarction or Ischemic Reperfusion

**DOI:** 10.14336/AD.2022.0416

**Published:** 2022-12-01

**Authors:** Ziyu An, Jinfan Tian, Yue Liu, Xin Zhao, Xueyao Yang, Jingwen Yong, Libo Liu, Lijun Zhang, Wenjian Jiang, Xiantao Song, Hongjia Zhang

**Affiliations:** ^1^Department of Cardiology, Beijing Anzhen Hospital, Capital Medical University, Beijing, China.; ^2^Cardiovascular disease center, Xiyuan Hospital, China Academy of Chinese Medical Sciences, Beijing, China.; ^3^Department of Cardiac Surgery, Beijing Anzhen Hospital, Capital Medical University, Beijing, China.

**Keywords:** cell-free therapy, exosome, acute myocardial infarction, ischemic reperfusion

## Abstract

Exosomes, which contain miRNA, have been receiving growing attention in cardiovascular therapy because of their role in mediating cell-cell communication, autophagy, apoptosis, inflammation, and angiogenesis. Several studies have suggested that miRNA derived from exosomes can be used to detect myocardial infarctions (MI) in patients. Basic research also suggests that exosomes could serve as a potential therapeutic target for treating acute myocardial infarction. Ischemia/reperfusion (IR) injury is associated with adverse cardiac events after acute MI. We aim to review the potential benefits and mechanisms of exosomes in treating MI and IR injury.

## 1. Introduction

Myocardial infarction (MI), the leading cause of death and disability worldwide, is commonly caused by a clot blocking an artery or bypass graft and is characterized by a sudden decrease in blood flow to the heart muscle, which can eventually lead to heart failure and death [1, 2]. Following MI, the decreased blood supply to the heart leads to apoptosis or necrosis of cardiomyocytes. Ischemia/reperfusion (I/R) injury, which results from endothelial inflammation, apoptosis, oxidative stress, and myocardial cell death, can cause ventricular remodeling and heart failure. Angiogenesis has an essential role in heart repair and the recovery of cardiac function, and impaired angiogenesis can suppress heart repair and the restoration of cardiac function. In recent years, various cell-secreted exosomes, such as adipose-derived MSCs, bone marrow mesenchymal stem cells (BMMSCs), and coronary serum exosomes, have been shown to alleviate myocardial injury after MI or I/R because of their anti-inflammatory, anti-oxidative stress, anti-apoptotic, anti-fibrotic, and pro-angiogenic properties. Thus, the use of exosomes has become a promising cell-free approach to cardiac repair following MI and I/R injury.

Exosomes, which are characterized as 40-100 nm diameter membrane-bound vesicles, can be found in almost all biological fluids. They are coated in a lipid bilayer and contain diverse biological molecules, including proteins, glycans, lipids, metabolites, RNA, and DNA [3-5]. After fusion with the plasma membrane, they are released from cells into the extracellular space. Lipids and proteins are the main constituents of the exosome membrane, which is rich in lipid rafts [6]. Additionally, a variety of nucleic acids, including mRNAs, microRNAs, and other non-coding RNAs (NC-RNA), have recently been discovered in exosome lumens [7]. When exosomes circulate, these exosome RNAs can be absorbed by adjacent or distant cells and subsequently modulate the recipient cells. Exosomes have received growing attention because of their function in genetic exchange between cells. The main function of exosomes may be related to RNA delivery. Exosomes appear to mediate cell-to-cell communication and play an essential role in both physiological and pathological processes [8]. Exosomes from donor cells mediate intercellular communication and trigger subsequent physiological alterations in recipient cells by binding to specific receptors, triggering membrane fusion and release. Important features of exosomes include the promotion of angiogenesis, tumor metastasis, dissemination of malignant tumors [9], alleviating I/R injury [10], and serving as antigen-presenting vesicles to stimulate T/B cells and induce cellular adaptive or humoral immunity [11].

## 2. Anti-inflammation and immune modulation

**Figure 1.**
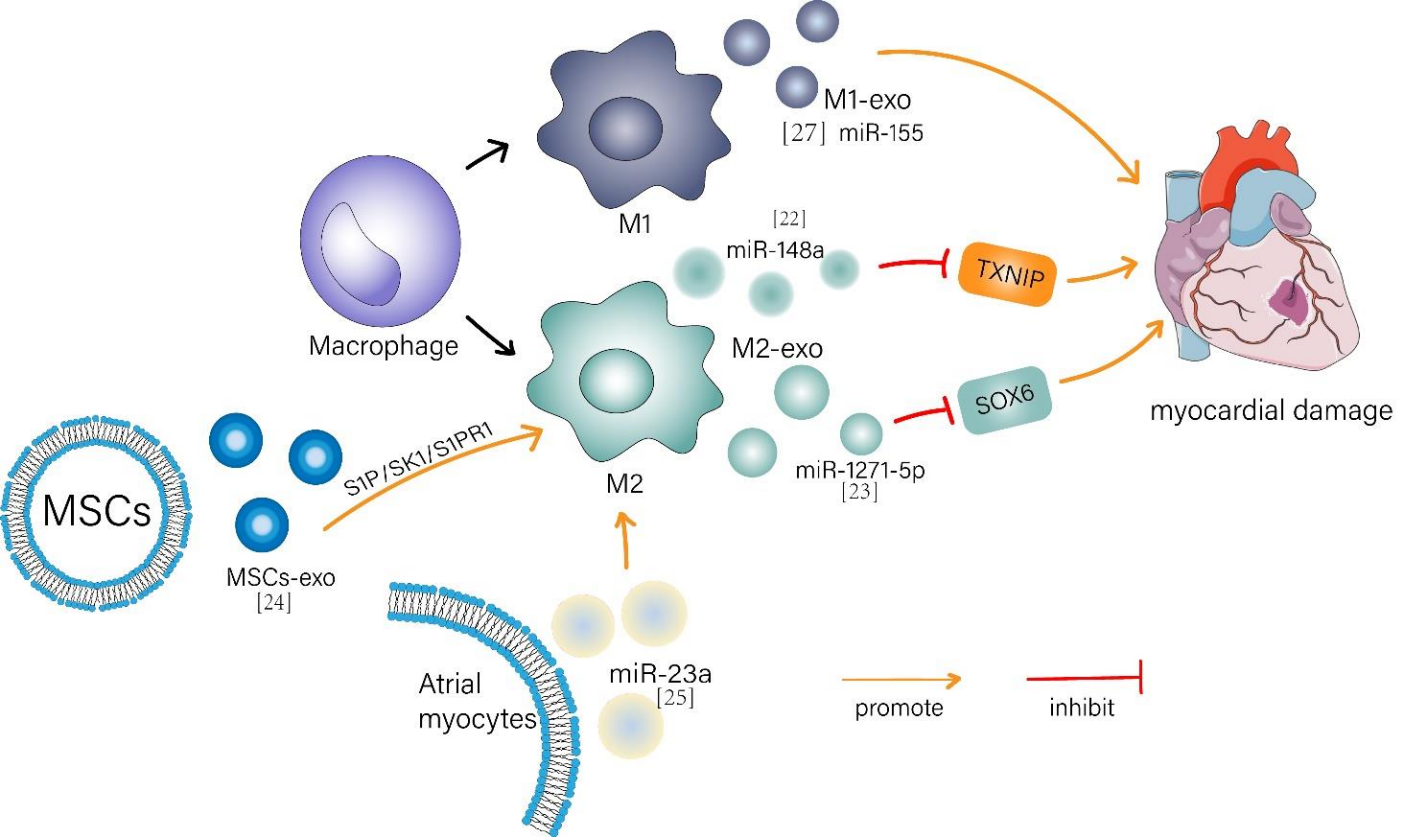
Partial anti-inflammatory properties of exosomes after MI or IR. Note: Exosomes produced by M2 macrophages (M2-exo) carrying miR-148a down-regulate thioredoxin-interaction protein (TXNIP) to reduce MI or I/R damage. In addition, M2-exo can carry miR-1271-5P to negatively regulate SOX6 expression and inhibit myocardial apoptosis. Mesenchymal stem cells derived exosomes (MSCs-exo) promote M2 polarization of macrophages and improve myocardial injury after myocardial infarction by activating the S1P/SK1/S1PR1 signaling pathway. Exosomes from Ang II-treated atrial myocytes promote the polarization of M2 macrophages by delivering miR-23a. In contrast, M1-Exo overexpressed inflammatory miR-155, aggravating the myocardial injury.

It is well-established that inflammation promotes myocardial injury following MI and I/R jury. Evidence suggests that nearly one in five patients with MI are affected by severe systemic inflammatory complications and are at a high risk of death [12, 13]. Table 1 and Figure 1 show the relationship between certain exosome properties and the promotion of anti-inflammation following MI or IR.

Systemic inflammation is characterized by high levels of circulating pro-inflammatory cytokines, including TNFα, and low concentrations of anti-inflammatory cytokines, such as IL-10 [14]. Yujia Y et al.[15] found that IL-10 deficiency/inflammation altered the function, content, and therapeutic efficiency of endothelial progenitor cell (EPC)-derived exosomes by up-regulating integrin-linked kinase (ILK) enrichment in exosomes and activating the ILK-mediated NFκB pathway. In contrast, down-regulation of ILK in exosomes attenuates NFκB activation and reduces inflammation. In addition, Adipose-derived stromal cells (ADSCs) derived exosome that containing miR-93-5p can prevent myocardial injury through TLR4-mediated inflammatory responses [16]. Zilun W et al.[17]suggested that miR-181a may suppress inflammation and increase the proportion of regulatory T cells (Treg) cells by inhibiting the c-FOS protein. Administration of miR-181a from human umbilical cord blood-derived MSCs-exo effectively treated myocardial I/R injury, which was attributed to the inhibitory effects of immune miR-181a and the cellular targeting properties of MSCs-exo.

**Table 1:** The properties of exosomes in anti-inflammation following MI or I/R.

Model	Exosome type	Dosage	Mechanism	Related-miRNA	Involved pathway	Ref.
MI mice with LAD ligation	EPC-exo	50 μg in PBS	Reducing inflammatory response	-	NFκB	[15]
MI rats with LAD ligation	ADSCs-exo	400 μg in 200 μl PBS	Inhibiting inflammatory response	miR-93-5p	Atg7	[16]
MI mice with LAD ligation	Exosomes secreted by human umbilical cord blood-derived MSCs	-	Inhibiting inflammatory response	miR-181a	-	[17]
MI mice with LAD ligation	BMMSCs-exo, L-exo	50 μg in 50 μl PBS	Promoting M2 macrophage polarization	-	AKT1/AKT2	[20]
NCMs with H/R model, I/R rats with LAD ligation	M2-exo	-	Inhibiting Inflammation signaling pathway	miR-148a	TXNIP, TLR4/NF-κB/NLRP3	[22]
MI mice with LAD ligation	M2-exo	-	Regulating miR-1271-5p/SOX6 pathway	miR-1271-5p	miR-1271-5p/SOX6	[23]
MI mice with LAD ligation	ADMSC-exo	2.5 × 10^12 particles suspended in 500 μL PBS)	Promoting M2 macrophage polarization	-	S1P/SK1/S1PR1	[24]
MI mice with LAD ligation	Treg-exo	50 μg in 200 μlPBS	Promoting macrophage M2 polarization	-	TNF-α	[26]

Note: MI, myocardial infarction; H/R, hypoxia/reoxygenation; LAD, Left, anterior descending artery; MSCs: mesenchymal stem cells; EPC: endothelial progenitor cell; L-exosomes, lipopolysaccharide pre-conditioning; exo, exosome; BMMSCs, bone marrow mesenchymal stem cells; M2-exo, M2 macrophages-derived exosome; ADSC: adipose-derived stromal cells; ADMSCs, adipose-derived MSCs; NCMs, neonatal rat cardiomyocytes; TXNIP, thioredoxin-interacting protein; Treg, regulatory T cells.

### 2.1 Targeting Macrophages polarization

Management of macrophage polarization is an important pathway for exosomes to exert their anti-inflammatory effects. Macrophages are categorized into two groups: M1-like phenotypic macrophages, which secrete large amounts of the inflammatory cytokine’s tumor necrosis factor-α (TNF-α), interleukin 1β and interleukin 6, and m2-like phenotypic macrophages that produce anti-inflammatory cytokines (interleukin 10, IL-10). M1 macrophages are recruited into the infarcted myocardium during the early stages of MI; in the later stage, M2 macrophages are recruited for anti-inflammatory activity later. Thus, the balance of M1 and M2 macrophages could be a potential target for MI and I/R injury treatment. Previous studies have shown that MSCs-derived exosomes have anti-inflammatory functions, which they exert by converting pro-inflammatory M1 to anti-inflammatory M2 macrophages [18, 19]. Ruqin Xu et al. [20] showed that both BMMSCs-derived exosomes and lipopolysaccharide (LPS)-pretreated BMMSCs (L-exosomes) increased the polarization of M2 macrophages and decreased M1 polarization under LPS stimulation. AKT is upstream of the NF-kb pathway and macrophages polarization pathway. AK1 largely regulates the polarization of M2 macrophages, while AKT2 mainly regulates M1 macrophage polarization [21]. AKT1 and AKT2 knockdown attenuated L-exosome’s effects on macrophage polarization, indicating that the AKT1/AKT2 pathway contributed to L-exosome-regulated macrophage polarization [20].

Yuxiang D et al. [22] found that via suppression of thioredoxin- interaction protein (TXNIP) and the TLR4/NF-κB/NLRP3 inflammatory vesicle signaling pathway, exosomes produced by M2 macrophages (M2-exos) carried miR-148A into neonatal rat cardiomyocytes (NCMs) and attenuated MI or I/R injury. In addition, M2-exos promotes cardiac repair following AMI by inhibiting cardiomyocyte apoptosis via negatively regulating SOX6 expression, a result of M2-exos derived miR-1271-5p suppression of cardiomyocyte apoptosis via the miR-1271-5p/SOX6 pathway [23]. Shengqiong D et al. [24] showed that adipose-derived MSCs (ADMSC) exosomes promoted the polarization of M2 macrophage, while the suppressive effects of ADMSC exosomes on myocardial apoptosis and fibrosis induced by MI were reversed by sphingosine-1-phosphate receptor 1 (S1PR1). M2 macrophage polarization induced by ADMSC-exosomes was also reversed after down-regulation of S1RP1. Together, these results suggest that exosomes ameliorate cardiac injury after MI by stimulating the S1P/SK1/S1PR1 signaling pathway and facilitating macrophage M2 polarization. Exosomes derived from atrial myocytes treated with Ang II promote the polarization of M2 macrophages by transferring miR-23a [25]. Hao Hu et al. [26] showed that treg-derived exosomes promoted M2 polarization in RAW264.7 cells in vitro and that Treg-derived exosomes injected into the anterior wall of the left ventricle in AMI mice inhibited cardiomyocyte apoptosis and improved AMI cardiac function by downregulating the expression of M1 macrophage markers (TNF-α, IL-1β, and IL-6) and promoting M2 macrophage markers (TGF-b1 and IL-10).

In contrast, Shaojun L et al.[27] found that inflammatory m1-like macrophages accelerate MI injury by releasing a large number of exosomes that promote inflammation (M1-EXOs). M1-Exos showed high expression levels of inflammatory miR-155, which translocated to endothelial cells (ECs) and suppressed angiogenesis and cardiac dysfunction by down-regulating Sirtuin 1 (Sirt1), p21 (RAC1)-activated kinase 2 (PAK2), Rac family small GTPase 1 (RAC1), and protein kinase AMP-activated catalytic subunit alpha 2 (AMPKα2). Additionally, M1-exos aggravated the myocardial injury and inhibited cardiac healing by reducing ECs angiogenesis (via inhibition of the Sirt1/AMPK α2 endothelial nitric oxide synthase and RAC1-PAK2 signaling pathways).

### 2.2 Immune modulation

Liu et al.[28] harvested MI-DEXs (exosomes isolated from bone marrow dendritic cells) from mouse spleens following DEXs injection and found that MI-DEXs injections could promote the recovery of cardiac function after MI by mediating CD4+ T cell activation through endocrine mechanisms. In another experiment [29], the same group found that miR-494-3p, which promotes angiogenesis, was significantly up-regulated and highly enriched in DEX in the MI group compared to the control group and that DEX-miR-494-3p enhanced angiogenesis in mice after MI. These findings suggest a potential for a new DEX-based treatment for MI.

Balanced inflammatory processes mediated by multiple immune cells and inflammatory factors play a key role in myocardial necrosis and repair after MI. Thus, modulating the inflammatory process after MI may be a potential therapeutic avenue. Exosomes, as key mediators of intercellular dialogue, effectively modulate immune cells and immune responses after MI, facilitating the repair process of the infarcted myocardium and maintaining ventricular function and cardiomyocytes through communication between immune cells [30].

## 3. Anti-oxidative stress

Oxidative stress due to myocardial injury contributes to cardiomyocyte apoptosis, but attenuating oxidative stress contributes to reduced myocardial apoptosis and fibrosis [31]. Jing N et al. [32] showed that exosomes extracted from human umbilical cord mesenchymal stem cells (HucMSCs) limited extracellular matrix (ECM) remodeling. Additionally, exosomes derived from TIMP2-overexpressing hucMSCs (HucMSCs-exo^TIMP2^) increased superoxide dismutase (SOD) and glutathione (GSH) while reducing malondialdehyde (MDA) in MI model mice. In vitro, Huc-exo^TIMP2^ pretreatment inhibited H2O2-mediated apoptosis of H9c2 cardiomyocytes, promoted human umbilical vein endothelial cell (HUVEC) proliferation, migration and tube formation, and reduced the secretion of α-SMA, MMP2, and MMP9 by cardiac fibroblasts (CFs) stimulated by TGF β. In addition, HucMSCs-exo^TIMP2^ pretreatment increased Akt phosphorylation level in infarcted myocardium, suggesting that HucMSCs-exo^TIMP2^ alleviates MI-induced oxidative stress and ECM remodeling, improving cardiac function partly through the Akt/Sfrp2 pathway.

Myocardial I/R injury is commonly accelerated by decreased ATP production, elevated oxidative stress and apoptosis, and cell death. MSC-derived exosomes carry all five enzymes of the glycolytic ATP production phase, along with phosphorylated PFKFB3, which up-regulates rate-limiting glycolytic enzymes. In I/R rats, MSC-derived exosomes contribute to reduced infarct sizes and adverse remodeling by increasing tissue ATP production in the heart, which could supplement depleted cellular antioxidants in I/R myocardium because it contains peroxidase and glutathione S-transferase. Additionally, MSC-derived exosomes decreased pro-apoptotic phosphorylation of c-JNK by increasing AKT and GSK2 phosphorylation [33].

In one study by Chang C et al. [34], circulating exosomes were obtained from AMI patients (AMI-exo) and healthy controls (normal-exo). In vitro and in vivo, circulating exosomes have been shown to confer protection against oxidative stress on HUVECs. At the same time, normal-exo had better protective effects. Highly expressed in normal exosomes, miR-193a-5p protects endothelial cells in vitro and in vivo by targeting a potential downstream molecular Activin A receptor type I (ACVR1). Activation of ACVR1 attenuated the protective effects of miR-193A-5p. However, ACVR1 inhibitors have protective effects that are similar to those of miR-193A-5p. These findings suggest that circulating exosomes protect endothelial cells from oxidative stress by targeting ACVR1 and delivering miR-193A-5p. Table 2 shows the anti-oxidative properties of exosomes following MI or IR.

**Table 2:** The properties of exosomes in anti-oxidative stress following MI or I/R.

Model	Exosome	Dosage	Mechanism	Related-miRNA	Involved Pathway	Ref.
MI mice with LAD ligation H9c2 treated with H2O2	hucMSCs-exo^TIMP2^	50 μg/ml	Anti-oxidative stress	-	Sfrp 2/AKT	[32]
I/R mice with LAD ligation	MSCs-exo	0.4 μg/ml MSC-exo	Anti-oxidative stress	-	PI3K/AKT	[33]
Patients who were 3-7 days after AMI	Normal-exo, AMI-exo	-	Anti-oxidative stress	miR-193a-5p	ACVR1	[34]

Note: MI, myocardial infarction; LAD, left anterior descending artery; AMI, acute myocardial infarction; MSCs: mesenchymal stem cells; hucMSCs: human umbilical cord mesenchymal stem cells; ACVR1, activin A receptor type I; HucMSCs-exo^TIMP2^, exosomes derived from TIMP2-overexpressing hucMSCs; normal-exo, exosomes from healthy controls; AMI-exo, exosomes from AMI patients.

## 4. Reducing cell death

Inhibiting cardiomyocyte cell death can improve cardiac dysfunction and help reverse adverse myocardial remodeling. Apoptosis under pathophysiological conditions (i.e., inflammation and oxidative stress, as well as excess autophagy) contribute to cardiomyocyte death following MI [35]. As mentioned above, exosomes could attenuate cardiomyocyte apoptosis via anti-inflammatory and anti-oxidative stress pathways (Table 3). Additionally, accumulating studies have demonstrated that exosomes reduce cell death by targeting autophagy [36] (Table 4).

Some exosomes can reduce myocardial cell death through multiple pathways, including anti-inflammatory, anti-oxidative stress, and anti-apoptotic pathways. Xue W et al. [37] investigated the role of exosomes secreted by ADMSCs (ADMSCs-exo) in MI progression. miR-671 was significantly up-regulated following exosome treatment, while the downregulation of miR-671 diminished the protective properties of exosomes. The results indicate that exosomal miR-671 reduced myocardial cell apoptosis, myocardial fibrosis, and inflammation by targeting transforming growth factor receptor 2 (TGFBR2) and inhibiting the phosphorylation of Smad2. Junjie Pan et al. [38] showed that miR-146a interacts with the 3 '-untranslated region of EGR1 to inhibit the expression of post-transcriptional EGR1. Inhibition of EGR1 expression reversed the activation of AMI and hypoxia-induced TLR4/NFκB signaling, suggesting that miR-146a modifies adipose-derived stem cells (ADSCs)-derived exosomes and could thus inhibit AMI-induced apoptosis, inflammation, and fibrosis. Qiancheng L et al. [39] also showed that miR-126-overexpressing ADSCs-derived exosomes prevented myocardial injury by protecting myocardial apoptosis, inflammation, and fibrosis, as well as by increasing angiogenesis.

### 4.1 Anti-apoptosis

**Figure 2.**
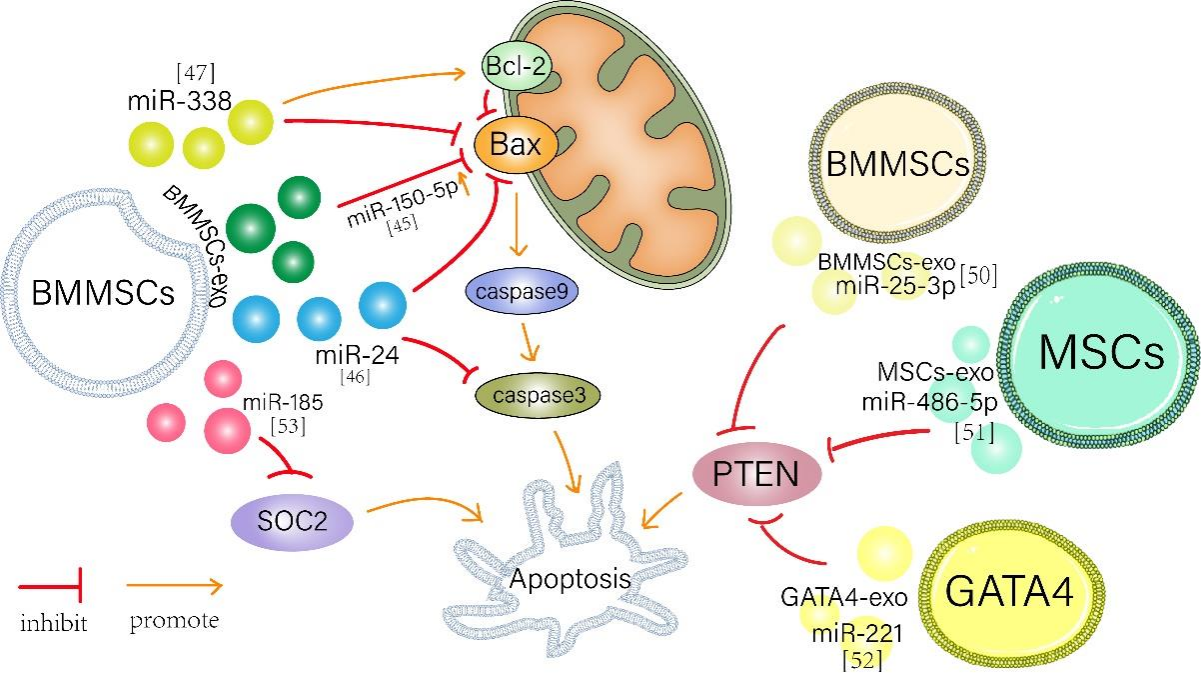
Partial anti-apoptosis properties of exosomes after MI or IR. Note: Bone marrow mesenchymal stem cells derived exosomes (BMMSCs-exo) increased miR-150-5p expression and decreased Bax expression in MI mice. The level of miR-24 was up-regulated, and the protein expressions of Bax and Caspase-3 were down-regulated. Overexpression of miR-338 by BMMSCS-exo decreased the expression of Bax, increased the expression of Bcl-2, and significantly reduced apoptosis. The expression of miR-25-3p in bone marrow mesenchymal stem cells derived exosomes (BMMSCS-exo) targets the pro-apoptotic gene PTEN and reduces its protein level. MiR-486-5p in bone marrow stromal cell-derived exosomes inhibits PTEN expression. In addition, miR-221 secreted by GATA4-exo down-regulated PTEN expression and reduced apoptosis. BMMSCs-exo expression of miR-185 reduces SOCS2 expression to protect against myocardial apoptosis.

Apoptosis is a type of programmed death (another way that necrosis leads to cell death). Structural changes occur in apoptotic cells, including cell shrinkage, nuclear pyknosis, and fragmentation [40]. Studies have shown that myocardial apoptosis begins with short-term ischemia following long-term myocardial ischemia or reperfusion[41]. Apoptosis begins at the edge of the infarct area within hours to days after acute infarction [42]. Growing numbers of studies showed exosomes restore the survival of cells and cardiac function by protecting against cardiomyocyte apoptosis (Table 3 and Fig. 2).

Bax is an important pro-apoptotic protein in the Bcl-2 family, and its stability is critical for regulating mitochondrial apoptotic pathways [43]. Bax is also involved in myocardial apoptosis in MI [44]. Zheng W et al. [45] demonstrated that the myocardial tissue of MI mice showed decreased expression of miR-150-5p and increased Bax expression, and BMMSCs-exo was associated with increased miR-150-5p expression and decreased Bax expression in MI mice. These findings indicate that exosome miR-150-5p can improve cardiac function in MI mice by reducing myocardial pathological alterations and decreasing apoptosis via the targeting of Bax. Zhang et al. [46] showed that miR-24 mRNA levels in the hypoxic preconditioning BMMSCs-exo group were higher than in the normoxic preconditioning BMMSCs-exo group. miR-24 levels were significantly up-regulated in AMI rats via injection of hypoxic BMMSCs-exo, reducing infarct size and improving cardiac function. Bax, caspase-3, and cleaved caspase-3 expression was down-regulated by hypoxic preconditioning BMMSCs-exo therapy. miR-24 levels were increased, and the H9c2 cell apoptotic rate was decreased after hypoxic preconditioning with BMMSCs-exo pretreatment. Fu et al. [47] showed that Bax and caspase 3 decreased, but Bcl-2 expression increased when H9c2 cells were co-cultured with rat BMMSCs-exo that overexpressed miR-338, which led to a significantly reduced H9c2 apoptosis rate. The cardiac function of rats was significantly improved after intramuscular injection of exosomes overexpressing miR-338. They further demonstrated that exosomal miR-338 could suppress myocardial cell apoptosis in MI rats and improve myocardial function by regulating the JNK pathway through the targeting of MAP3K2.

Tumor suppressor phosphatase and tensin homology (PTEN) is also associated with apoptosis [48, 49]. PTEN down-regulation can regulate cell proliferation and promote cell apoptosis. Yi Peng et al.[50] showed that exocytosis of BMMSCs resulted in a significant increase in miR-25-3p in cardiomyocytes, which directly reduced the expression of the pro-apoptotic genes FASL and PTEN. miR-25-3p has also been shown to diminish the levels of Enhancer of zest homolo2 (EZH2) and H3K27me3, which leads to down-regulation of the cardioprotective gene eNOS and the anti-inflammatory gene SOCS3. MSC co-culture can reduce OGD-induced cardiomyocyte apoptosis and inflammatory responses, driving the overexpression of miR-25-3p. MiR-486-5p in bone marrow stromal cells derived-exosomes inhibited the H/R triggered apoptosis of H9c2 cells. Bone marrow stromal cells derived- exosomes inhibited the expression of PTEN in H9c2 cells via miR-486-5p and also activated the PI3K/AKT pathway in vitro. In vivo, the PI3K/Akt pathway can repair myocardial I/R injury [51]. Chunshu Hao et al. [52] also showed that miR221, which targets PTEN, was increased when H9c2 cells were treated with GATA4-exo. GATA4-exo decreased apoptosis of H9c2 cells by down-regulating PTEN expression.

**Table 3:** The properties of exosomes in reducing cell apoptosis following MI or I/R.

Model	Exosome type	Dosage	Mechanism	Related-miRNA	Involved Pathway	Ref.
MI mice with LAD ligation	ADMSCs-exo	100 μg in PBS	Anti-apoptosis, and anti-inflammation	miR-671	TGFBR2	[37]
MI mice with LAD ligation	ADSCs-exo	400 μg in 200 μl PBS	Anti-apoptosis, and anti-inflammation	miR-146a	EGR1 TLR4/NFκB	[38]
MI mice with LAD ligation	ADSCs-exo	400 μg in 200 μl PBS	Anti-apoptosis, and anti-inflammation	miR-126	-	[39]
MI mice with LAD ligation	BMMSC-exo	-	Anti-apoptosis	miR-150-5p	Bax	[45]
MI rats with LAD ligation, H9c2 cells with H2O2	BMMSCs-exo	-	Anti-apoptosis	miR-24	Bax, caspase-3	[46]
MI rats with LAD ligation	BMMSCs-exo	50 μl	Anti-apoptosis	miR-338	JNK	[47]
I/R mice with LAD ligation	BMMSCs-exo	5 μl in 100μl PBS	Anti-apoptosis, and anti-inflammation	miR-25-3p	EZH2 FASL	[50]
H9c2 cells with H/R treatment	bone marrow stromal cells derived exosomes	400 μg in 200 μl PBS	Anti-apoptosis	miR-486-5p	PI3K/AKT	[51]
MI mice with LAD ligation	GATA4-exo	100 μg in 100 μl PBS	Anti-apoptosis	miR-221	PTEN/PI3K/AKT	[52]
MI mice with LAD ligation	BMMSC-exo	5 μg in 25μl solution	Anti-apoptosis	miR-185	SOCS2	[53]
MI mice with LAD ligation, H9c2 cells with hypoxia	hucMSCs-exo	400 μg/g	Anti-apoptosis	miR-19a	SOX6	[54]
MI mice with LAD ligation	hucMSCs-exo and OMSCs-exo	1 × 10^6 MSCs in 30 μl PBS	Anti-apoptosis	miR-136	Apaf1	[55]
MI mice with LAD ligation	Hypo-BMMSCs-exo	-	Anti-apoptosis	miR-125b	P53, BAK1	[57]
MI rats with LAD ligation	BMMSCs-exo	-	Anti-apoptosis	miR-133	-	[58]
NRCMs incubated in a thermostatic and hypoxic condition	BMMSCs, ADMSCs, and UCBMSCs derived exosomes	25 μg/ml	Anti-apoptosis	-	VEGF	[60]
MI mice with LAD ligation	ADRCs-exo	-	Anti-apoptosis	miR-214	-	[62]
MI mice with LAD ligation	GATA-4-BMMSCs-exo	20 μg/ml	Anti-apoptosis	miR-233	GATA-4	[64]
MI mice with LAD ligation	BMMSCs-exo	-	Anti-apoptosis	miR-210	AIFM3	[66]

Note: MI, myocardial infarction; LAD, Left anterior descending artery; MSCs: mesenchymal stem cells; ADMSCs, adipose-derived MSCs; ADSCs, adipose-derived stem cells; TGFBR2, transforming growth factor receptor 2; BMMSCs: bone marrow mesenchymal stem cells; UMSCs: human umbilical cord MSCs; OMSCs: bone marrow MSCs from the old person; Hypo-BMMSC-exo: BMMSCs under hypoxia; UCBMSCs: umbilical cord blood-derived mesenchymal stem cells; NRCMs, neonatal rat cardiomyocytes; Apaf1: apoptotic peptidase activator; ADRCs: adipose-derived regenerative cells-derived exosomes; PTEN, phosphatase and tensin homology.

Exosomes can alleviate MI by inhibiting apoptosis through other pathways. Yanbing Li et al. [53] found that BMMSCs-Exo-driven expression of miR-185 promoted myocardial functioning in MI mice, alleviated myocardial injury, and protected against myocardial cell apoptosis by decreasing SOCS2 expression. Overexpression of SOCS2 blocked the cardioprotective effects of BMMSCs-exo derived miR-185 in MI mice. Lin Huang et al. [54] found that exosomes from hucMSCs could alleviate AMI and inhibit myocardial cell apoptosis. Knockdown of miR-19a in hucMSC-exo appears to attenuate the protective effect of hucMSC-exo against AMI injury. Inhibition of SOX6, a target gene of miR-19a, attenuated hypoxic injury in H9c2 cells. In hucMSC-exo, miR-19a inhibited SOX6-activated AKT and suppressed the JNK3/caspase-3 axis to protect cardiomyocytes from AMI injury. Ning Zhang et al. [55] found that the expression of miR-136 in huMSCs and bone marrow MSCs from (OMSCs)-exo from elderly patients was higher than that in OMSCs. Up-regulation of miR-136 significantly reduced apoptosis and senescence of OMSCs, and apoptotic peptidase activator (Apaf1) was negatively regulated by miR-136 via the direct targeting of its 3'UTR. The exosomes from young MSCs can improve the activity of aged MSCs and enhance myocardial repair mechanisms in in MI mice by transferring exosomes miR-136 and down-regulating apoptotic peptidase activator (Apaf1). Xiaolin Liu et al. [56] demonstrated that MSC-derived exosomes mediate cardiac repair after MI. Under hypoxia/serum deprivation stimulation, neonatal mice cardiomyocytes (NRCMs) that were treated by exosomes isolated from BMMSCs transducted with Macrophage migration inhibitory factor (MIF) plasmid (MIF-BMMSCs-exo) showed less apoptosis than those that were treated with BMMSCs-exo via the activation of adenosine 5 '-monophosphate-activated protein kinase (AMPK). MIF-BMMSC-exo enhanced cardiac function, reduced cardiac remodeling, and reduced reactive oxygen species production and apoptosis. Lingping Zhu et al. [57] found that exosomes secreted by BMMSCs under hypoxia (hypo-exo) had protective effects against ischemic disease, and miR-125b-5P was significantly enriched in hypo-exo. miR-125b knockdown of hypo-exo significantly increased infarct size and inhibited myocardial cell survival after MI. Mechanically, miR-125b knockdown hypo-exo attenuated its ability to inhibit the expression of the pro-apoptotic genes p53 and BAK1 in cardiomyocytes. Yueqiu Chen et al. [58] found that transfection with miR-133 resulted in a significant reduction in hypoxia-induced apoptosis and enhanced expression of total poly ADP-ribose polymerase protein in BMMSCs. In vivo, MI rats transplanted with miR-133-MSCs exhibited better improvement in cardiac function and reduced infarct MI size after the inhibition of cardiac SNAIL1 expression [59].

There are also some exosomes with unspecific signaling pathways that may or may not inhibit apoptosis. In a study by Huiyu Xu et al. [60], exosomes derived from BMMSCs, ADMSCs, and umbilical cord blood-derived mesenchymal stem cells (UCBMSCs) inhibited the apoptosis of neonatal rat cardiomyocytes incubated with 0.5% O2 and 5% CO2 for 24h. Exosomes produced by Cardio sphere-derived cells (CDCs) can reduce cellular apoptosis. In a study by Helia Namazi et al. [61], exosomes produced by CDCs under normoxic (18% O2) and hypoxic (1% O2) conditions could inhibit cell apoptosis, and the anti-apoptotic activity of exosomes in normoxic states at the concentrations of 25 and 50 μg/mL was greater than that in a hypoxic state. Shunsuke Eguchi et al.’s experiments [62] demonstrated that exposure of cardiomyocytes to adipose-derived regenerative cells (ADRCs)-derived exosomes (ADRC)-exo prevented cardiomyocyte damage under hypoxic conditions in vitro, while silencing miR-214 in ADRC-exo significantly impaired the anti-apoptotic effects of ADRC-exo treatment on cardiomyocytes both in vitro and in vivo. C-X Li et al. [63] showed that exosomes from UMSCs also inhibited apoptosis of myocardial cells and promoted MI repair by delivering CIRC-0001273. Jigang He et al. [64] showed that GATA4-BMMSCs exosomes injected 48h after MI enhanced cardiac function for the following 96h. Vascular density and c-kit positive cell number increased, and myocardial cell apoptosis decreased. The co-culture of GATA4-BMMSCs exosomes with cardiomyocytes under hypoxia can reduce cellular apoptosis and improve myocardial function after infarction. Yi-Qing Z et al. [65] extracted exosomes from human amniotic epithelial cells (HAEC) and established a stable AMI model in rats. They studied the changes in cardiac function following treatment with HAEC, exosomes, or PBS. The results showed that the rates of cardiomyocyte apoptosis were significantly reduced in the HAEC group and exosome group. Hao C et al. [66] found that AIFM3 was a downstream target of miR-210 expressed in BMMSCs, and treatment of miR-210 overexpressed BMMSC exosomes could improve the protection of cardiomyocytes against in vitro and in vivo stress. Shuo Wang et al. [67] showed that, in vitro, miR-19A/19b inhibited cardiac HL-1 cellular apoptosis using exosomes from BMMSC as carriers. In vivo, Exo/miR-19A/19b on its own or in combination with MSC transplantation significantly promoted the recovery of cardiac function and attenuated myocardial fibrosis in an MI model.

On the contrary, some microRNA appears to promote MI injury. Jie Chun Huang et al. [68] found that miR-328-3p was significantly increased in MI cardiomyocytes and neighboring normal cardiomyocytes. Exogenous exosome miR-328-3p enhances apoptosis and MI injury in cardiomyocytes. Those genes regulated by miR-328-3p are mainly found in the Caspase signaling pathway. miR-328-3p is a novel potential diagnostic biomarker and therapeutic target for MI, while Z-DevD-FMK can reverse the miR-328-3p-induced apoptotic process.

### 4.2 Targeting autophagy

Autophagy is an essential metabolic process in which aged or damaged proteins and organelles are catabolized into amino acids and fatty acids, which are then recycled and used for energy production [69]. In the presence of nutritional deficiencies or increased metabolic stress, these metabolic processes are activated to maintain tissue function and dynamic homeostasis [70]. Moderate autophagy activation does not lead to cell death in myocardial ischemia-reperfusion injury and maybe a protective mechanism [71]. However, under the influence of different signaling pathways, both up-regulation and down-regulation of autophagy may be protective to the myocardium [72]. The following experiments demonstrated the protective effects of exosome-promoted autophagy on the myocardium (Table 4).

**Table 4:** The properties of exosomes in targeting autophagy following MI or I/R.

Model	Exosome type	Dosage	Mechanism	Related-miRNA	Involved Pathway	Ref.
MI rats with LAD ligation	MSCs-exo	-	Promoting autophagy	miR-301	AMPK/mTOR Akt/mTOR	[73]
MI mice with LAD ligation	VECs-exo	-	Inhibiting autophagy	-	Akt/AMPK	[74]
MI rats with LAD ligation	BMMSCs-exo	-	Inhibiting autophagy	miR-301	-	[75]
MI rats with LAD ligation H9c2 cells with 1% O2, 94% N2, and 5% CO2	BMMSCs-exo	200 μg in 400 μl PBS	Anti-apoptosis inhibiting autophagy	miR-143-3p	CHk2-Beclin2	[77]
MI rats with LAD ligation	ADSCs-exo	400 μg in 200μl PBS	Inhibiting autophagy	miR-93-5p	Atg7	[16]
MI mice with LAD ligation	BMMSCs-exo	5 μg in 25 μl solution	Inhibiting autophagy	miR-125b	BAFA1 LC3-II	[78]

Note: MI, myocardial infarction; LAD, left anterior descending artery; MSCs: mesenchymal stem cells; VECs: vascular endothelial cells; ADSCs, adipose-derived stem cells; BMMSCs, bone marrow mesenchymal stem cells.

Liang Liu et al. [73] demonstrated that MSC-derived exosomes reduced H2O2-induced H9c2 apoptosis by enhancing autophagy 12 h after exposure. In vivo, MSC-exo reduced MI size by up-regulating autophagy through the AMPK/mTOR and Akt/mTOR pathways, which consequently led to an up-regulated expression of myocardial LC3B and improved cardiac function. In other conditions, inhibition of autophagy may also confer a protective effect on the myocardium. Qiang Su et al. [74] showed that exosomal LINC00174 produced by vascular endothelial cells directly interacts with SRSF1 to inhibit p53 expression, thereby inhibiting both cardiomyin transcription and the activity of the Akt/AMPK pathway. The Akt/AMPK pathway plays a key role in autophagy initiation in I/R induced myocardial injury. Exosomal LINC00174 produced by vascular endothelial cells inhibits p53-mediated autophagy and apoptosis, which alleviates I/R induced myocardial injury. MI rats treated with BMMSCs-exo transfected with miR-301 mimics showed significantly increased left ventricular ejection fractions, left ventricular fractional shortening and p62 expression in MI tissues, decreased left ventricular end-systolic diameter, decreased left ventricular end-diastolic diameter (LVEDD), decreased MI area, decreased microtubule-associated protein 1 light chain 3 (LC3) II/LC3-I ratios, and decreased numbers of autophagosomes [75]. Chang Zhang et al. [76] showed that epigallocatechin gallate (EGCG) enhanced cardio-myocyte activity and also increased the concentration, average diameter, miR30a mRNA levels, and expression of specific proteins in AMI-derived exosomes that are generated by cardiomyocytes. Co-incubation of AMI cells with exosomes derived from EGCG or cardiomyocytes treated with EGCG reduced autophagy and apoptosis. Gecai Chen et al. [77] found that BMMSCs and BMMSCs-derived exosomes suppress the apoptosis and autophagy induced by H/R. The CHk2-Beclin2 pathway has a role in H/R-induced autophagy, while miR-143-3p negatively regulates CHK2 expression in a direct manner. Hence, exosomal miR-143-3p protects the myocardium by inhibiting H/R-induced autophagy and targeting the CHk2-Beclin2 pathway. Jiwen Liu et al. [16] found that inflammatory cytokines and miR-93-5p levels were elevated in both AMI patients and animal models. ADSC-derived exosomes containing miR-93-5p have a greater protective effect on myocardial injury induced by infarction via decreases in autophagy. In vitro, miR-93-5p expression can target Atg7 and Toll-like receptor 4 (TLR4), respectively, and inhibit the expression of hypoxia-induced autophagy and inflammatory factors. Changchen Xiao et al. [78] showed that infarct size and cardiac function measurements were significantly better in the BMMSCs-exo group than in the BMMSCs-exo anti-miR-125b group. Treatment with transplanted MSCs-exo containing miR-125b improved myocardial function and reduced infarct size by reducing apoptosis and autophagic flux (BAFA1-induced LC3-II accumulation and autophagosome/autophagic lysosomal prevalence) in the infarcted myocardium of mice.

## 5. Anti-fibrosis

Following myocardial infarction, the proliferation of fibroblasts causes the formation of noncontractile scar tissue, which can compromise cardiac function [79]. Myocardial fibrosis commonly results from irreversible myocardial tissue damage that is caused by MI[80]. Xiaoke et al. [81] showed that exo-miR-218-5p or exo-miR-363-3p, which is enriched in endothelial progenitor cell-derived exosomes (EPC-Exos), promotes mesenchymal endothelial cell transformation and inhibits myocardial fibrosis by up-regulating p53 and down-regulating the expression of a junction-mediated regulatory protein. Lianmei Pu et al. [82] showed that miR-30e expression was poor in the myocardial tissue of MI rats. Exo overexpression of miR-30e can improve pathological damage, apoptosis, and fibrosis in rat myocardial tissue. miR-30e negatively regulates LOX1 expression, while further exosome treatment inhibits LOX1 expression. In addition, Exo overexpression of miR-30e impaired the NF-κB P65/Caspase-9 signaling pathway in the myocardial tissue of MI rats, which inhibited myocardial apoptosis and fibrosis. Jingyu Deng et al. [83] showed that hypoxic stress could lead to increased apoptosis and decreased cell viability, while fibronectin type III domain-containing protein 5 (FNDC5-OV) could alleviate this damage. FNDC5-MSC treatment significantly reduced cardiac fibrosis and alleviated cardiac dysfunction. Interestingly, FNDC5-OV increased exosome secretion from BM-MSCs. Warren D. Gray et al. [84] demonstrated that cardiac progenitor cells (CPCs)-exosomes secreted under hypoxic conditions facilitated tubular formation by endothelial cells and that exosome injection reduced the expression of pro-fibrotic genes in fibroblasts stimulated by TGF-β. Exosomes from hypoxic CPCs improved cardiac function and reduced fibrosis after I/R injury. Jianan L et al. [25] concluded that exosomes derived from atrial myocytes treated with Ang II inhibit the polarization of M2 macrophages and inhibit the expression of markers of atrial fibroblast fibrosis by transferring miR-23a. Collectively, these studies indicated that exosomes protected against MI or I/R injury via anti-fibrosis pathways (Table 5).

**Table 5:** The properties of exosomes in anti-fibrosis following MI or I/R.

Model	Exosome type	Dosage	Mechanism	Related-miRNA	Involved Pathway	Ref.
MI mice with LAD ligation	ADMSCs-exo	100 μg in PBS	Anti-fibrosis	miR-671	TGFBR2	[37]
MI mice with LAD ligation	ADSCs-exo	400 μg in 200μl PBS	Anti-fibrosis	miR-146a	EGR1/TLR4/NFκB	[38]
MI mice with LAD ligation	ADSCs-exo	400 μg in 200μl PBS	Anti-fibrosis	miR-126	-	[39]
MI rats with LAD ligation	EPCs-exo	300 μg in 150μl PBS	Anti-fibrosis	miR-218-5p	-	[81]
MI rats with LAD ligation	BMMSCs-exo	20 μg/ml	Anti-fibrosis	miR-30e	NF-κB P65/Caspase-9	[82]

Note: MI, myocardial infarction; LAD, left anterior descending artery; MSCs: mesenchymal stem cells; ADMSCs, adipose-derived MSCs; ADSCs, adipose-derived stem cells; TGFBR2, transforming growth factor receptor 2; BMMSCs, bone marrow mesenchymal stem cells; EPCs: endothelial progenitor cells

## 6. Angiogenesis

Angiogenesis, or the process of forming new capillaries on the basis of existing microvasculature, depends on the migration and proliferation of vascular endothelial cells and is essential for repairing the ischemic microenvironment [85]. Exosomes protect against MI/IR by promoting angiogenesis (Table 6). Vascular endothelial growth factor (VEGF) is the key factor driving cardiac angiogenesis following MI[86]. VEGF genes could significantly improve myocardial perfusion, increase vascular density and ventricular function [87]. Hypoxia-inducible factor-1α (HIF-1α) is an upstream factor in the regulation of VEGF that is induced by umbilical cord mesenchymal stem cells (ucMSCs) and thus plays an important role in both angiogenesis and cardiac repair [88]. In MI rats, the injection of exosomes derived from BMMSCs, ADMSCs, and UCMSCs around the margin area of MI promotes angiogenesis by up-regulating VEGF[60]. Peisen Huang et al. [89] showed that exosomes from atorvastatin-pretreated MSCs (MSCATV-exo) administration prevented H/SD-induced endothelial cell apoptosis. MSCATV-exo promotes angiogenesis in the per-infarct area and inhibits IL-6 and TNF-α elevation.

**Table 6:** The properties of exosomes in angiogenesis following MI or I/R.

Model	Exosome type	Dosage	Mechanism	Related-miRNA	Involved pathway	Ref.
MI rats with LAD ligation	BMMSCs, ADMSCs, and UCBMSCs derived exosomes	150 μl of 1.5×10^6^ exosomes	Angiogenesis	-	VEGF	[60]
MI rats with LAD ligation	MSCATV-exo	10 μg in 100μl PBS	Angiogenesis	lncRNA H19	VEGF	[89]
MI mice with LAD ligation	DEXs	-	Angiogenesis	miR-494-3p	VEGF	[29]
MI rats with LAD ligation	BMMSCs-exo	-	Angiogenesis	-	VEGF	[90]
MI rats with LAD ligation	hucMSCs-exo^TIMP2^	50 μg/ml	Angiogenesis	-	Sfrp 2/AKT	[32]
HUVECs treated with H2O2	hucMSCs-exo^TIMP2^	50μg/ml	Proliferation, migration	-	Sfrp 2/AKT	[32]
MI rats with LAD ligation	BMMSCs-exo	1 μg/μl	Angiogenesis,	miR-221-3p	Akt	[92]
MI rats with LAD ligation	HBO-induced exo	70 μg in 150 μl	Angiogenesis	miR-92a	KLF2	[95]
MI mice with LAD ligation	CPCexo-322	5.0 µg/ml	Angiogenesis	miR-322	-	[96]

Note: MI, myocardial infarction; LAD, left anterior descending artery; MSCs: mesenchymal stem cells; BMMSCs, bone marrow mesenchymal stem cells; ADMSCs, adipose-derived MSCs; UCBMSCs: umbilical cord blood-derived mesenchymal stem cells; MSCATV-exo, atorvastatin-pretreated MSCs; DEXs: exosomes isolated from bone marrow-derived dendritic cells; HUVECs, human umbilical cord vein endothelial cells; MALAT1, metastasis-associated lung adenocarcinoma transcript 1; hucMSCs-exoTIMP2, exosomes derived from TIMP2-overexpressing hucMSCs; HBO, hyperbaric oxygen; CPCexo-322: CPCexo transfected with a pro-angiogenic miR-322; KLF2: krüppel-like factor 2.

LncRNA H19 acts as a mediator for MSCATV-exo to regulate the miR-675 expression and the activation of VEGF and intercellular adhesion molecule-1. Haibo Liu et al. [29] showed that DEXs significantly enhanced the tubular capacity of cardiac microvascular endothelial cells (CMECs) by up-regulating the expression of VEGF, CD31, and miR-494-3p in CMECs. DEX-miR-494-3p enhanced CMECs tube formation and angiogenesis in mice after MI. Kai Kang et al. [90] showed that exosomes from BMMSC transduced with lentiviral CXCR4 (Exo(CR4)) promote cardiac function recovery, reduce infarct size, and improve cardiac remodeling by increasing angiogenesis by up-regulating VEGF. However, Akt inhibitors and CXCR4 knockdown eliminate the protective effects of Exo (CR4). According to Zhang et al., Wnt4 induces β-catenin activation in endothelial cells and exerts pro-angiogenic effects, and hucMSC-derived exosomes promote angiogenesis by regulating the Wn4/β-catenin signaling pathway, suggesting it may also be a potential target for exosome repairment [91]. Jing Ni et al. showed that hucMSCs-exo^TIMP2^ promotes HUVEC proliferation and migration, as well as the numbers and length of cell tubes formed in vitro. In vivo, administration of huc-exo^TIMP2^ in the peri-infarct zone in MI rats significantly increased the *in situ* expression of CD31[32]. In a study by Ling Sun et al. [92], young MSCs-exo were found to be superior to aged MSCs-exo in promoting intravascular formation, reducing fibrosis, and inhibiting apoptosis. Additionally, miR-221-3p was significantly downregulated in aged MSCs-exo. When miR-221-3p was overexpressed in aged Exos, aged MSCs were activated, and their capability to repair the heart was restored. miR-221-3p’s effects are realized by inhibiting PTEN to enhance Akt kinase activity. In a study by Hao Li et al. [93], exosomes from patients with myocardial ischemia (isc-Exo) were found to promote endothelial cell proliferation, migration, and catheter formation. In a mouse hindlimb ischemia model, perfusion and histological staining showed that isc-Exo significantly promoted the restoration of blood flow and angiogenesis by downregulating miR-939-5p, which was associated with diminished endothelial cell NO production and impaired angiogenesis. Hui Huang et al. [94] showed that exosomes derived from Sirtuin 1 (SIRT1)-overexpressing adipose-derived stem cells (ADSCs-SIRT1-exos) increased the expression of chemokine 12 and nuclear factor E2-related factor 2 in peripheral blood endothelial progenitor cells (EPCs) from AMI patients (AMI-EPCs). Moreover, ADSCs-SIRT1-exos could lead to the overexpression of C-X-C chemokine receptor type 7, which help AMI-EPCs to restore cell migration and tube formation. Collectively, ADSCs-SIRT1-exos are capable of improving survival rates, promoting the recovery of myocardial function, reducing infarct size and left ventricular remodeling, and inhibiting myocardial inflammation caused by AMI. Kou-gi Shyu et al. [95] found that exosomes from cardiac myocytes that were induced by hyperbaric oxygen (HBO) significantly increased the expression of metastasis-associated lung adenocarcinoma transcript 1 (MALAT1) in cardiomyocytes, which inhibited miR-92a expression, and attenuated the suppressive effects of miR-92a on krüppel-like factor 2 and CD31 expression in the left ventricular myocardium after MI, enhancing neovascularization. Seock-won Youn et al. [96] found that CPCexo transfected with a pro-angiogenic miR-322 (CPCexo-322) provided greater protection after ischemic injury in mice, in part by enhancing angiogenesis in the boundary region of the infarcted heart. Mechanically, CPCexo-322 treatment of cultured human endothelial cells (ECs) leads to a greater angiogenic response, as determined by increasing NADPH oxidase-derived ROS to increase EC migration and capillary formation. Ning Zhang et al. [97] also demonstrated that Mono-exos promoted endothelial maturation and regulated macrophage subsets during post-MI/RI angiogenesis and ultimately improved cardiac function and histopathological changes in MI/RI mice after treatment.

## 7. Perspective

Stem cell therapy has also received previous attention for its potential application in cardiac repair [98, 99]. Stem cells secrete cytokines and extracellular vesicles to regulate the process after MI. However, despite some successful results in animal models, results in clinical trials have been generally poor, mainly because of limited survival and retention of stem cells after transplantation [100]. Exosome therapy is now a possible alternative to cell therapy for the treatment of MI[101]

Exosomes is emerging as a cell-free therapy for ischemic myocardium and as serum targets for myocardial infarction [102]. Mei-Li Zheng et al. [103] showed that, in patients with AMI, lncRNAs ENST00000556899.1 and ENST00000575985.1 of circulating exosomes were elevated and thus represented potential biomarkers for predicting the prognosis of patients with AMI. According to Gabriella Andriolo et al. [104], Exo-CPC as a final product (GMP-Exo-CPC) can improve cardiac function after MI and can subsequently be applied to other cell sources in various therapeutic areas. Min Cheng et al. [105]showed that after the injection of exosomes isolated from AMI mice into wild-type mice, CXCR4 expression in bone marrow mononuclear cells was down-regulated, while the number of circulating progenitor cells was increased. These results suggest that myocardial microRNAs (MYO-miRs) carried in circulating exosomes allow for a systemic response to cardiac injury, which could be harnessed for cardiac repair. Combined delivery of exosomes and stem cells in a sequential manner may be effective in reducing scar size and restoring cardiac function after AMI [106]. Ischemic myocardial targeting peptide CSTSMLKAC (IMTP) produces exosomes that specifically target ischemic myocardium, and MSC-derived IMTP-exosomes have enhanced therapeutic effects in acute MI [107]. Through the endocrine mechanism, MI DEXs could mediate the activation of CD4+ T cells and improve cardiac function after myocardial infarction, which provides a basis for a new strategy of systemic delivery of DEXs for treating MI [28]. In a study by Zhenzhen Chen et al. [108], NEAT1, miR-204, and MMP-9 were shown to be effective biomarkers for diagnosing acute ST-segment elevation MI. Exosomes derived from BMMSCs expressing GATA-4 promote differentiation of MSCs into cardiomyocyte-like cells, reduce hypoxia-induced apoptosis and improve cardiac function after MI [64]. Compared to exosomes alone, the injection of exosome/PGN hydrogel mixture into the periphery of infarcted rat hearts improved myocardial function by reducing inflammation, fibrosis, and apoptosis and by promoting angiogenesis [109].

In conclusion, in this perspective, we have discussed the effects of various cellular exosomes on improving cardiac function in MI or I/R and the related mechanisms. We believe that exosome-based cell-free therapy is a promising approach for the treatment of acute MI and I/R injury.
